# Plasma PolyQ-ATXN3 Levels Associate With Cerebellar Degeneration and Behavioral Abnormalities in a New AAV-Based SCA3 Mouse Model

**DOI:** 10.3389/fcell.2022.863089

**Published:** 2022-03-21

**Authors:** Karen Jansen-West, Tiffany W. Todd, Lillian M. Daughrity, Mei Yue, Jimei Tong, Yari Carlomagno, Giulia Del Rosso, Aishe Kurti, Caroline Y. Jones, Judith A. Dunmore, Monica Castanedes-Casey, Dennis W. Dickson, Zbigniew K. Wszolek, John D. Fryer, Leonard Petrucelli, Mercedes Prudencio

**Affiliations:** ^1^ Department of Neuroscience, Mayo Clinic, Jacksonville, FL, United States; ^2^ Neurobiology of Disease Graduate Program, Mayo Graduate School, Mayo Clinic College of Medicine, Rochester, MN, United States; ^3^ Department of Neurology, Mayo Clinic, Jacksonville, FL, United States; ^4^ Department of Neuroscience, Mayo Clinic, Scottsdale, AZ, United States

**Keywords:** spinocerebellar ataxia 3, SCA3, Machado-Joseph disease, ATXN3, polyglutamine, mouse model, aav, biomarker

## Abstract

Spinocerebellar ataxia type 3 (SCA3) is a dominantly inherited cerebellar ataxia caused by the expansion of a polyglutamine (polyQ) repeat in the gene encoding ATXN3. The polyQ expansion induces protein inclusion formation in the neurons of patients and results in neuronal degeneration in the cerebellum and other brain regions. We used adeno-associated virus (AAV) technology to develop a new mouse model of SCA3 that recapitulates several features of the human disease, including locomotor defects, cerebellar-specific neuronal loss, polyQ-expanded ATXN3 inclusions, and TDP-43 pathology. We also found that neurofilament light is elevated in the cerebrospinal fluid (CSF) of the SCA3 animals, and the expanded polyQ-ATXN3 protein can be detected in the plasma. Interestingly, the levels of polyQ-ATXN3 in plasma correlated with measures of cerebellar degeneration and locomotor deficits in 6-month-old SCA3 mice, supporting the hypothesis that this factor could act as a biomarker for SCA3.

## Introduction

Spinocerebellar ataxia type 3 (SCA3), also known as Machado-Joseph disease, is the most common dominantly inherited cerebellar ataxia worldwide. Patients suffer from the dysfunction and degeneration of the cerebellum and spinocerebellar tracts, along with neuronal loss in other regions including the brainstem, midbrain, spinal cord, striatum, and thalamus (McLoughlin et al., 2020). This degeneration leads to a progressive loss of locomotor control and eventual demise. SCA3 is caused by the expansion of a CAG repeat in the gene ataxin-3 (*ATXN3*), which results in a polyglutamine (polyQ) expansion in the ATXN3 protein. Similar CAG expansions in other genes are associated with Huntington’s disease, spinal and bulbar muscular atrophy, dentatorubral-pallidoluysian atrophy, and additional spinocerebellar ataxias (SCA1, SCA2, SCA6, SCA7, and SCA17). In all of these diseases, the polyQ expansion renders the host protein aggregation-prone and toxic, and the native functions of each host protein are believed to influence the resulting patterns of selective degeneration in each condition (Orr, 2012). As *ATXN3* knockout mice fail to show overt abnormalities (Schmitt et al., 2007), it is hypothesized that the mutant ATXN3 protein confers disease through a toxic gain of function, although the disruption of endogenous ATXN3 activity may also contribute (McLoughlin et al., 2020).

There are currently no effective therapeutics for SCA3, but gene therapies aimed at reducing mutant ATXN3 protein levels are gaining momentum (Miller et al., 2003; Alves et al., 2008; Alves et al., 2010; Evers et al., 2011; Nobrega et al., 2013b; Evers et al., 2013; Rodriguez-Lebron et al., 2013; Nobrega et al., 2014; Moore et al., 2017; McLoughlin et al., 2018; Ouyang et al., 2018). As these potential therapies and others move towards clinical trials, there is an increased need for adequate biomarkers that can both predict disease progression in SCA3 and provide an accurate readout of target engagement. Methods of detecting the mutant protein in patient biofluids are of interest, as are biomarkers associated with neurodegeneration like neurofilament light (NFL). Our group recently developed an immunoassay that can detect the expanded ATXN3 protein (polyQ-ATXN3) in the cerebrospinal fluid (CSF), plasma, and urine of SCA3 patients (Prudencio et al., 2020; Koike et al., 2021). We found that both NFL and polyQ-ATXN3 levels in patient biofluids can distinguish SCA3 patients from controls, supporting the use of these factors as diagnostic and pharmacodynamic biomarkers in clinical studies (Prudencio et al., 2020; Koike et al., 2021).

In addition to biomarkers, the development and preclinical testing of potential therapeutics also requires animal models. Several mouse models of SCA3 have been developed over the years, using both transgenic and knock-in approaches, and many of these models recapitulate the ataxia-like behavior, inclusion pathology, and cerebellar degeneration expected of SCA3 (Ikeda et al., 1996; Cemal et al., 2002; Goti et al., 2004; Bichelmeier et al., 2007; Chou et al., 2008; Torashima et al., 2008; Boy et al., 2009; Boy et al., 2010; Silva-Fernandes et al., 2010; Simoes et al., 2012; Nobrega et al., 2013a; Silva-Fernandes et al., 2014; Ramani et al., 2015; Switonski et al., 2015; Haas et al., 2021). But the utility of mouse models doesn’t stop there: researchers use mice to evaluate potential genetic modifiers and test mechanistic hypotheses. This work is often time-consuming and expensive, as it generally requires crossing the disease model to other mutant and knockout mouse lines. In this study we present an adeno-associated virus (AAV)-based mouse model of SCA3 that we believe will expedite future studies. AAV technology has been used to generate mouse models of other neurodegenerative diseases, including *C9orf72*-associated amyotrophic lateral sclerosis (ALS) (Chew et al., 2015; Chew et al., 2019), Alzheimer’s disease (Ittner et al., 2019), Huntington’s disease (Maxan et al., 2020) and SCA36 (Todd et al., 2020), and these models successfully recapitulate disease features. Our AAV model of SCA3 expresses full-length human ATXN3 with 84 glutamine repeats (Q84) throughout the mouse brain. We find that the expression of this mutant protein successfully induces ataxia-like behavioral defects that are not observed in control animals expressing a non-pathogenic repeat length of 28 glutamines (Q28). The mutant protein also forms inclusions in the brains of the mice and results in the selective loss of cerebellar Purkinje cells. In congruence with SCA3 patient studies (Li et al., 2019; Prudencio et al., 2020), mice expressing the pathogenic repeat protein showed an increase in NFL levels in the CSF and an accumulation of polyQ-ATXN3 in the plasma. Higher levels of plasma polyQ-ATXN3 associated with a thinner cerebellar molecular layer thickness and decreased mobility in 6-month-old mice, suggesting this potential biomarker could correlate with disease phenotypes.

## Materials and Methods

### AAV Preparation

The coding sequences for myc-ATXN3 with 28 or 84 polyQ repeats (Addgene plasmids #22125 and #22124, respectively (Chai et al., 1999); both gifts from Dr. Henry Paulson, University of Michigan) were cloned into the AAV expression vector pAM/CBA-WPRE-BGH. AAV particles were packaged into the serotype 9 type capsid and purified using standard methods (Zolotukhin et al., 1999). Briefly, AAV was generated by co-transfection with the cis plasmid pCap9 (a gift from Dr. Todd Golde, University of Florida) into HEK 293T/17 cells (ATCC #Crl-11268). Cells were harvested 72 h after transfection, treated with 50 units/ml benzonase endonuclease (Sigma-Aldrich), and lysed by freeze-thaw. The virus was then purified using a discontinuous iodixanol gradient and buffer exchanged to phospho-buffered saline (PBS) using an Amicon Ultra-15 Centrifugation Filter Unit Ultracel-100 (Millipore #UFC9110024). The genomic titer of each virus was determined by quantitative PCR (qPCR) using the Quant Studio 7 Flex Real-Time PCR System (Applied Biosystems), Sybr Green PCR Master Mix (Applied Biosystems #4309155), and primers specific to the WPRE. Samples were compared against a standard curve of supercoiled plasmid diluted to 1 × 10^4^ to 1 × 10^7^ copies per ml.

### Animal Husbandry

All animal studies were performed in accordance with the National Institutes of Health Guide for Care and Use of Experimental Animals and approved by the Mayo Clinic Institutional Animal Care and Use Committee (protocol #A00001780-16-R19). Animals were maintained in standard housing on a 12-h light/dark cycle with access to water and food *ad libitum*. All mice were of the same genetic background (C57BL/6 J) and divided into experimental groups based on the AAV received. Ideal total sample size was determined by power analysis, and approximately 15–20 postnatal day 0 (P0) pups were injected per group to ensure that both males and females were sufficiently represented in the final adult cohorts. As sex cannot be determined at P0, exact numbers of males and females were not equal in the resulting cohorts, but no differences were observed between males and females, except for expected differences in overall body weight. Total *n* numbers for each cohort were as follows: 3 months Q28: *n* = 15 (6 males and 9 females). 3 months Q84: *n* = 24 (8 males and 16 females). 6 months Q28: *n* = 16 (5 males and 11 females). 6 months Q84: *n* = 16 (10 males and 6 females).

### Neonatal Injections

Intracerebroventricular (ICV) injections of AAV were performed as previously described (Chew et al., 2015; Chew et al., 2019). Briefly, each AAV solution was diluted to a concentration of 1.5 × 10^10^ genomes/μl. C57BL/6 J pups at P0 were cryoanesthetized on ice for approximately 3 min or until they exhibited no movement. Two microliters of either AAV2/9-mycATXN3-Q28 or AAV2/9-mycATXN3-Q84 solution was manually injected into each cerebral ventricle using a 32-gauge needle (Hamilton Company) attached to a 10 μl syringe (Hamilton Company). The needle was inserted at a 30-degree angle from the surface of the head at a point approximately two-fifths the distance between the lambda suture and the eye and held at a depth of approximately 2 mm when injecting. After injections, the pups were placed on a heat pad until they completely recovered from anesthesia; they were then returned to their home cages.

### Behavioral Analyses

Mice expressing either AAV2/9-mycATXN3-Q28 or AAV2/9-mycATXN3-Q84 were aged to either 3 or 6 months before undergoing a battery of behavioral tests, including the hindlimb clasping test, ledge test, wire hang test, rotarod test, and open field assay. All mice were acclimated to the testing room for 1–2 h prior to testing. All behavioral equipment was cleaned with 30% ethanol prior to use with each animal. All mice were returned to their home cages and home room after each test. Researchers performing the behavioral analyses were blinded to the identity of the mice (Q28 versus Q84).

#### Hindlimb Clasping Test

Mice were lifted from their cage by the base of their tails and hindlimb position was observed for 10 s. Hindlimb clasping behavior was scored for each mouse according to a standard rubric for cerebellar ataxia models (Guyenet et al., 2010). If the hindlimbs were splayed outward, the mouse received a score of 0. If a single hindlimb was retracted toward the abdomen for more than 50% of the time suspended, the mouse was assigned a score of 1. If the hindlimbs were partially retracted for more than 50% of that time, it was assigned a score of 2, and if the hindlimbs were completely retracted for more than 50% of the time suspended, the mouse received a score of 3. This test was repeated 3 times and each mouse was assigned an average score.

#### Ledge Test

The ledge test was performed according to established methods (Guyenet et al., 2010). Each mouse was lifted from their home cage, placed on the ledge of an empty cage, and assigned a score depending on how it lowered itself into the test cage. A score of 0 was assigned if the mouse lowered itself into the test cage using its paws, a score of 1 if the mouse lost its footing or seemed uncoordinated, a score of 2 if it descended into the cage without using its hind legs, and a score of 3 if it fell off the ledge or would not walk or lower itself into the cage. This test was repeated 3 times and each mouse was assigned an average score.

#### Wire Hang Test

A 55 cm wide and 2 mm thick plastic-coated wire was secured to 2 vertical stands 35 cm above a layer of soft pads. Each mouse was suspended from the tail and lowered towards the wire, allowing the animal to grasp the wire with its forefeet. The mouse was then allowed to hang from the wire until it fell onto the soft padding below. The time until this first fall was recorded as the latency to fall. Each mouse was then returned to the wire and the total number of falls over a 2-min testing period was recorded. This test was repeated 3 times per mouse and the scores were averaged.

#### Rotarod Test

Motor coordination and balance were measured using an automated rotarod system (Med Associates, Inc.). Each mouse was placed on an accelerating spindle (starting at 4 rpm and increasing up to 40 rpm) for 5 min for 3 consecutive trials with at least 20 min of rest in between trials. When each mouse fell off the spindle, it triggered a sensor that automatically stopped a timer located underneath the spindle. This time was recorded as the latency to fall. If a mouse clung to and rolled around the rod 3 consecutive times, the timer was stopped, the mouse was manually removed from the spindle, and the corresponding time was recorded. This test was repeated for four consecutive days.

#### Open Field Assay

The apparatus consists of a square Perspex box (40 cm × 40 cm x 30 cm, L x W x H) with side mounted photobeams raised 7.6 cm above the floor to measure rearing. Mice were placed in the center of an open field area and were allowed to explore the area for 15 min. Movement was monitored through the use of an overhead camera with AnyMaze software (Stoelting Co.). Mice were tracked for multiple parameters, including total distance traveled, average speed, time mobile, and distance traveled in an imaginary “center” zone (20 cm × 20 cm), which was illuminated by an overhanging light fixture. This “center zone” was further subdivided into a small center and the total “big” center. Anxiety levels in the mice were measured by comparing the distance traveled in the “center zone” vs the total distance traveled: mice with higher anxiety levels show less exploratory behavior and prefer to stay near the outer walls of the apparatus. Consequently, these animals spend less time in the “center zone”.

### Tissue and Biofluid Collection

After behavioral analyses, mice expressing either AAV2/9-mycATXN3-Q28 or AAV2/9-mycATXN3-Q84 were anesthetized with 90–120/10 mg/kg of ketamine/xylazine through intraperitoneal administration. The depth of anesthesia was evaluated by toe and tail pinch, and when the mice were fully sedated, the skin and fur covering the top of head and neck of the mice were removed to reveal the subcutaneous tissue and muscles underneath. Muscles overlaying the magna cisterna were cut and moved to the side, revealing the dura mater. To prevent contamination of the CSF during collection, the area was cleansed with cotton swabs soaked in sterile PBS and dried to remove any blood. Excessive bleeding around the area was ceased by a cauterizer pen. A 26 5/8-gauge needle was used to gently penetrate the magna cisterna through the dura mater, and CSF was collected with a gel loading tip. CSF was transferred into protein low binding tubes (Eppendorf) and immediately frozen on dry ice. The volume of CSF collected per mouse ranged from 8 to 15 µl. Blood plasma samples were collected by cardiac puncture and initially stored on wet ice before being spun at 5,000 rpm for 10 min at 4°C, transferred to a new tube, and frozen on dry ice. After sample collection, mice were euthanized and the brain was harvested and bisected at the sagittal midline. One hemibrain was regionally dissected and immediately frozen on dry ice, while the other hemibrain was fixed in 4% paraformaldehyde for 48 h before being rinsed with PBS and embedded in paraffin. Fixed hemibrains were sectioned into 5 μm midsagittal slices, mounted on positively charged glass slides, and dried overnight. These paraformaldehyde-fixed, paraffin-embedded (FFPE) brain sections were then used in subsequent immunohistochemistry (IHC) analyses as described below.

Fresh-frozen forebrain and cerebellum tissues were later homogenized in TE buffer (50 mM Tris, pH 7.4, 50 mM NaCl, 1 mM EDTA) using 5 μl of buffer per milligram of tissue. These tissue homogenates were stored at -80 °C until used for RNA extraction or polyQ-ATXN3 immunoassays as described below.

### qPCR Analyses

To generate RNA lysates from the Q28 and Q84 mouse brains, 90 μl of forebrain or cerebellar TE homogenate was combined with 270 μl of Trizol LS Reagent (Ambion #10296010). RNA was extracted using the Direct-zol RNA Microprep Kit (Zymo Research #R2062) according to the manufacturer’s instructions. The total RNA concentration was measured using a UV spectrophotometer and cDNA was transcribed from 500 ng of RNA using the High Capacity cDNA Reverse Transcription Kit (Applied Biosystems #4374966), according to the manufacturer’s instructions. Two microliters of cDNA diluted 1:20 in H_2_O was used in a qPCR reaction using Sybr Green PCR Master Mix (Applied Biosystems #4309155) and detected on the Quant Studio 7 Flex Real-Time PCR System (Applied Biosystems) according to the manufacturer’s instructions. Mouse *Gapdh* was used as an endogenous control. The following forward/reverse primer pairs were used: human *ATXN3* (TAT​CGC​ACG​TTT​TTA​CAG​CAG​C/GCC​TCT​GAT​ACT​CTG​GAC​TGT​T), mouse *Atxn3* (GGA​GGA​TGA​AGA​AGC​TGA​TCT​C/TGG​ACT​TGA​TGT​CTG​TGG​ACT​ATT), mouse *Kcnc3* (TTT​TTG​AGG​ACC​CCT​ACT​CG/ATG​AAG​CCC​TCG​TGT​GTC​TC), mouse *Gapdh* (CAT​GGC​CTT​CCG​TGT​TCC​TA/CCT​GCT​TCA​CCA​CCT​TCT​TGA​T).

### NFL Immunoassay

NFL concentrations in the mouse CSF were measured in duplicate on a Simoa HD-X analyzer using an NF-Light digital immunoassay (Quanterix #103186) according to the manufacturer’s instructions. Briefly, samples were thawed and cleared by centrifugation at 10,000 x g for 5 min. Samples were diluted 1:25 at the bench, transferred to a 96-well plate, and ran in duplicate using a 4x instrument dilution for a final dilution of 1:100. NFL concentrations were interpolated from the calibration curve using a 1/y^2^ weighted four-parameter logistic curve fit.

### Immunohistochemistry and Analysis

FFPE brain sections from Q28 and Q84 mice were subjected to IHC staining as previously described (Chew et al., 2015; Chew et al., 2019). Sections were deparaffinized in xylene and rehydrated through a series of ethanol solutions. Antigen retrieval was performed by steaming in 1 mM sodium citrate pH 6.0/0.05% Tween 20 buffer for 30 min. Tissues were blocked in 2% normal goat serum in PBS, then immunostained with rabbit α-ATXN3 (1:200, clone 13HCLC, ThermoFisher #711823) or rabbit α-TDP-43 pS409/410 (1:500, rb3655, developed in-house) overnight at 4 °C. After washing, sections were incubated with an anti-rabbit secondary antibody at room temperature for 1 h. Peroxidase labeling was visualized with the chromogen solution 3,3′-diaminobenzidine (DAB-Plus), counterstained with Lerner 1 hematoxylin (Thermo Fisher Scientific) and coverslipped with Cytoseal mounting medium (Thermo Fisher Scientific). To stain with α-polyQ (1:1000, clone 5TF1-1C2, Millipore #MAB1574), α-ubiquitin (1:55,000, clone Ubi-1 042691 GS, Chemicon/Millipore #MAB1510), and α-NeuN (1:5,000, Chemicon International), slides were stained using the DAKO Autostainer (universal Staining System) and the DAKO + HRP system. Sections were then counterstained with hematoxylin, dehydrated through a series of ethanol and xylene washes, and mounted with Cytoseal mounting media (Thermo Fisher Scientific, Inc.). A similar protocol was used to detect ubiquitin (clone Ubi-1 042691 GS, Chemicon/Millipore #MAB1510) and polyQ (clone 5TF1-1C2, Millipore #MAB1574) in the brain of a SCA3 patient from the Mayo Clinic Brain Bank.

Slides were scanned with a ScanScope AT2 (Leica Biosystems) at either 20x or 40x magnification and images were analyzed using ImageScope and Aperio ePathology software (Leica Biosystems). The cerebellum was analyzed as previously described using serial sections stained with hematoxylin and eosin (H&E) (Chew et al., 2015; Todd et al., 2020). The number of Purkinje cells in each hemisection was counted manually and the length of the Purkinje cell layer was measured using ImageScope. The width of the molecular layer was measured using ImageScope at multiple locations for each fissure and averaged for each mouse. Data shown is from the posterolateral fissure (fpl). For NeuN analysis, the cortex or motor cortex was selected as a region of interest and the number of NeuN-positive cells per area was quantified using an algorithm designed to detect nuclei. All animals from each cohort were included, and researchers were blinded to the identity of the AAV received by each animal while performing these analyses.

### PolyQ-ATXN3 Protein Immunoassay

To generate protein lysates, 50 μl of forebrain homogenate was combined with 100 μl of CoIP Buffer (50 mM Tris-HCl, pH 7.4; 300 mM NaCl; 5 mM EDTA; 0.1% Triton-X-100) and adjusted to a final concentration of 2% SDS. The lysates were sonicated and total protein concentration was determined by BCA assay. An equal amount of total protein (5 μg) in CoIP SDS buffer was used per sample. These forebrain tissue lysate samples were then subjected to an immunoassay that measures polyQ-ATXN3 protein levels using Meso Scale Discovery (MSD) electrochemiluminescence detection technology, as described previously (Prudencio et al., 2020). To perform this assay, we coated MSD Multi-array 96-well plates (MSD #L15XA-6) with a mono-clonal anti-ATXN3 antibody (Millipore #MAB5360) overnight at a 1:2000 dilution: this serves as our capture antibody. Wells were washed with TBS-T (0.2% Tween 20 in TBS) and blocked with 3% MSD Blocker A (MSD #R93AA-1) in TBS-T at room temperature for 1 h. Wells were then washed 3 times with TBS-T and the forebrain tissue lysate samples were loaded in duplicate onto the assay plate (50 μl per well). The plates were incubated at room temperature at 600 rpm for 2 h, then washed again 3 times with TBS-T. For detection, an anti-polyQ detection antibody (clone 3B5H10, Sigma #P1874) was conjugated to a SULFO-TAG NHS-Ester group according to the manufacturer’s recommendation (MSD #R91AO-1) and used at 1.25 μg/ml. The plates were incubated for 1 h at room temperature at 600 rpm, after which time the plates were washed 3 times again with TBS-T and read with 1X MSD Read Buffer T with Surfactant (MSD #R92TC-2) on the MSD Meso QuikPlex SQ 120 Plate Reader. The same protocol was used to measure polyQ-ATXN3 levels in the plasma of the mice; plasma samples were diluted 1:4 in Diluent 12 (MSD #R50JA-3) and 50 μl were loaded per well. The CSF was not analyzed for polyQ-ATXN3 levels as we did not recover enough sample from each mouse to run both the NFL assay and this assay on the same cohort.

### Western Blot Analysis

Cell lysates were generated as described above, diluted with 2x SDS-loading buffer at a 1:1 ratio (v/v) and heated at 95 °C for 5 min. Equal amounts of protein were ran on 20-well 4–20% Tris-glycine gels (Novex) and proteins were transferred to PVDF membranes. Non-specific sites were blocked with 5% nonfat dry milk in TBS plus 0.1% Tween 20 for 1 h and the blots were then incubated with anti-ATXN3 (clone 13HCLC, ThermoFisher #711823) or anti-Gapdh (Meridian Life Science #H86504 M) antibody overnight at 4 °C. Membranes were washed three times in TBST and incubated with donkey anti-rabbit or anti-mouse IgG antibodies conjugated to horseradish peroxidase (1:5,000; Jackson ImmunoResearch) for 1 h. Membranes were washed again, and protein expression was visualized by enhanced chemiluminescence treatment and exposure to film. Bands were quantified using Scion Image by analyzing pixel density, and protein levels were normalized to Gapdh as the protein loading control.

### Statistical Analysis

Statistical analyses were performed in GraphPad Prism and both the test used and the definition of statistical significance applied are included in the figure legends. Unless otherwise noted, most analyses comparing the Q28 and Q84 cohorts at both time points were analyzed using 1-way ANOVA with Tukey’s post-hoc multiple comparisons test. Rotarod data was analyzed by 2-way ANOVA with Sidak’s post-hoc multiple comparisons test. Analyses comparing only two data sets were analyzed with an unpaired *t* test. All error bars represent the standard error of the mean (SEM).

## Results

### Generation of an AAV-Based SCA3 Model

**FIGURE 1:**
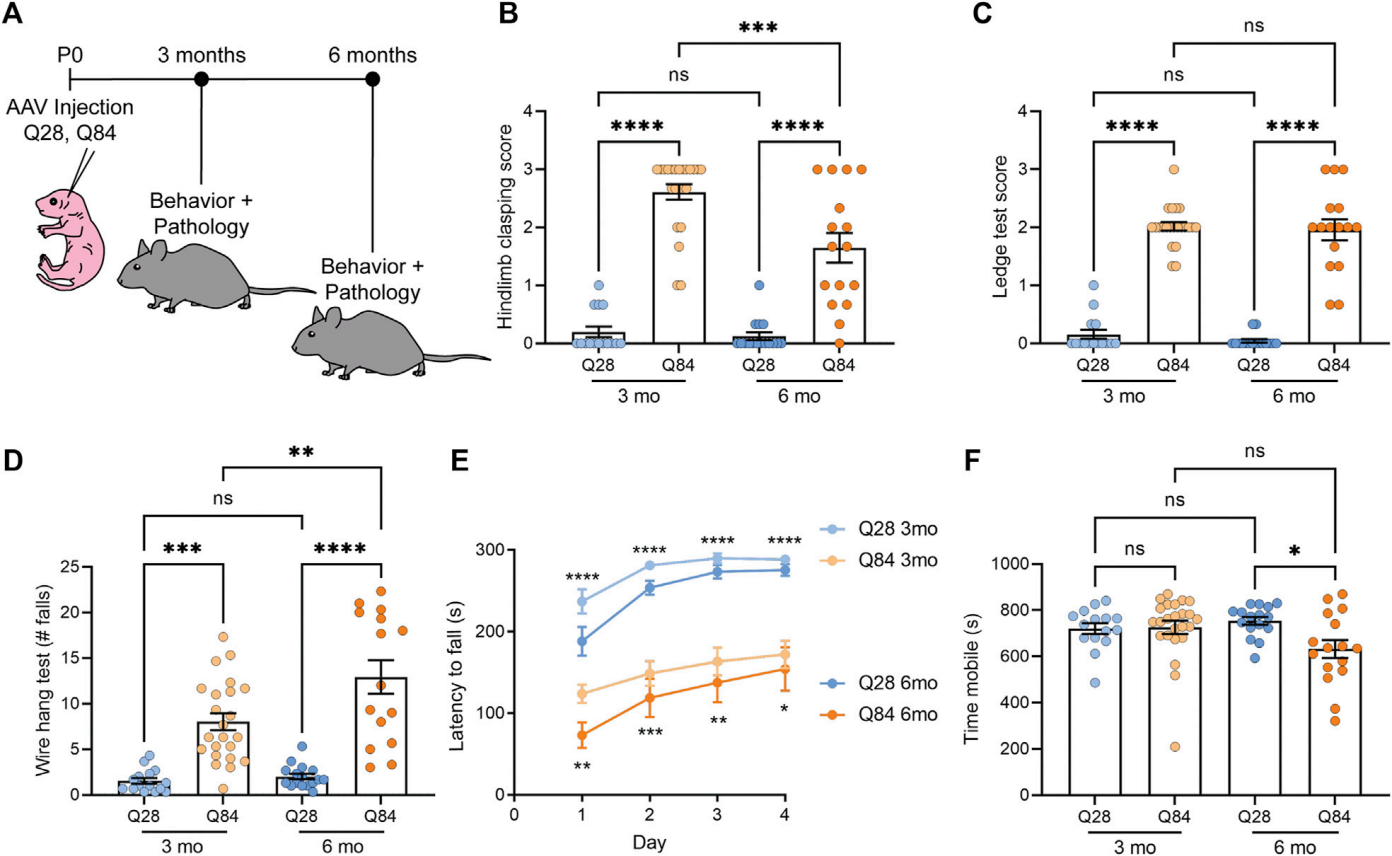
SCA3 mice show ataxia-like behavioral defects. **(A)** Schematic showing the experimental timeline used to analyze the Q28 and Q84 mice. AAV was injected at P0 and mice were analyzed for both behavior and pathology at 3 or 6 months. **(B)** Hindlimb clasping scores for Q28 and Q84 animals at 3 and 6 months of age. **(C)** Ledge test scores for Q28 and Q84 animals at 3 and 6 months of age. **(D)** Number of falls in 2 min on the wire hang assay for Q28 and Q84 animals at 3 and 6 months of age. **(E)** Latency to fall (seconds) on the rotarod over four consecutive days for Q28 and Q84 animals at 3 and 6 months of age. Asterisks at the top of the graph refer to analysis of Q28 versus Q84 in the 3-months cohort, while asterisks at the bottom of the graph refer to the 6-month-old animals. **(F)** Time (seconds) spent mobile during the open field assay for Q28 and Q84 animals at 3 and 6 months of age. For all panels, error bars are the standard error of the mean (SEM). *p ≤ 0.05, **p ≤ 0.01, ***p ≤ 0.001, ****p ≤ 0.0001, ns = non-significant (1-way ANOVA with Tukey’s multiple comparisons test for **(B**-**D**,**F)**; 2-way ANOVA with Sidak’s multiple comparisons test for **(E)**.

We subcloned myc-tagged human *ATXN3* with either 28 (normal, Q28) or 84 (pathogenic, Q84) CAG repeats into an AAV expression vector, packaged the constructs into AAV serotype 9 vectors, and injected them into the brains of mice at P0. This approach results in predominantly neuronal transduction in several regions of the mouse brain (Chakrabarty et al., 2013; Chew et al., 2015; Cook et al., 2015; Chew et al., 2019; Todd et al., 2020). Mice were aged to 3 or 6 months before being evaluated for behavioral defects. Mouse brains were then analyzed for pathology and molecular changes at both time points (Figure 1A). Expression of human *ATXN3* in both the forebrain and cerebellum of the mice was confirmed using qPCR. RNA levels were lower in the cerebellum than in the forebrain, but still clearly expressed (Supplementary Figure S1A). We also measured mouse *Atxn3* levels by qPCR and found that expression of the expanded polyQ-ATXN3 did not affect the expression of endogenous *Atxn3* in either brain region (Supplementary Figure S1B).

### SCA3 Mice Show Ataxia-like Behavioral Defects

Q84 and Q28 mice were weighed and subjected to a battery of behavioral tests to determine whether they showed locomotor defects. No significant difference in body weight was observed between the Q84 and Q28 animals (Supplementary Figures S2A,B). At both time points, Q84 mice showed a robust hind limb clasping defect (Figure 1B) and impaired performance on the ledge test (Figure 1C). The Q84 animals also fell sooner (Supplementary Figure S2C) and more often in 2 min (Figure 1D) when subjected to the wire hang test and showed impaired performance in the rotarod test (Figure 1E). By 6 months of age, the animals were generally less mobile in the open field assay (Figure 1F). Together, these results suggest that the Q84 mice showed defects in balance and locomotion—phenotypes akin to ataxia. The Q84 mice also showed a decrease in the center-to-total distance ratio in the open field assay (Supplementary Figures S2D,E), which could suggest some mild anxiety defects.

### SCA3 Mice Show Neuronal Loss Specifically in the Cerebellum

**FIGURE 2:**
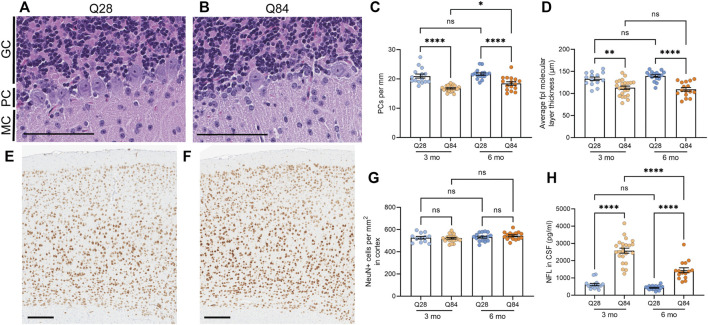
SCA3 mice show neuronal loss in the cerebellum and increased NFL levels in the CSF. **(A**–**B)** Representative images of the cerebellar Purkinje cell layer in Q28 **(A)** and Q84 **(B)** mice at 6 months of age. Slides were stained with H&E. MC: molecular cell layer; PC: Purkinje cell layer; GC: granular cell layer. Scale bars are 100 µm. **(C)** Average number of Purkinje cells (PCs) per mm of the Purkinje cell layer of the cerebellum in Q28 and Q84 animals at 3 and 6 months of age. **(D)** Average molecular layer thickness at the posterolateral fissure (fpl) in the cerebellum of Q28 and Q84 animals at 3 and 6 months of age. **(E**–**F)** Representative images of the cortex stained with NeuN in Q28 **(E)** and Q84 **(F)** mice at 6 months of age. Scale bars are 200 µm **(G)** Number of NeuN-positive cells per mm^2^ in the cortex of Q28 and Q84 animals at 3 and 6 months of age. **(H)** NFL levels in the CSF of Q28 and Q84 animals at 3 and 6 months of age. For all panels, error bars are the SEM. *p ≤ 0.05, **p ≤ 0.01, ***p ≤ 0.001, ****p ≤ 0.0001, ns = non-significant (1-way ANOVA with Tukey’s multiple comparisons test).

Following behavioral assessment at 3 and 6 months of age, mice were sacrificed and brains were harvested. The overall brain weight was decreased in the Q84 mice at both time points (Supplementary Figure S3A), potentially indicating neurodegeneration. Thus, we analyzed the brains for neuronal loss in the cerebellum, an area significantly affected in spinocerebellar ataxias. We noted a significant 15–20% decrease in the number of Purkinje cells per millimeter in the cerebellum of the Q84 mice compared to the Q28 controls (Figures 2A–C). We also noted a slight but significant decrease in the thickness of the cerebellar molecular layer, particularly at the posterolateral fissure (Figure 2D and Supplementary Figures S3B-E), suggesting possible degeneration in this region. These defects were detected at both time points analyzed. In contrast, when we measured the number of NeuN-positive cells per area in the cortex of these animals we saw no difference between the Q84 and Q28 mice (Figures 2E–G), even when we limited our analysis to the motor cortex (Supplementary Figure S3F). The Q84 animals therefore recapitulate the pattern of selective neuronal vulnerability associated with spinocerebellar ataxias like SCA3. Furthermore, we found that the number of remaining Purkinje cells per millimeter in the Q84 animals correlated with the severity of their behavioral defects: mice with fewer Purkinje cells showed worse performance in the ledge test (Supplementary Figure S4A), less exploratory behavior in the open field assay (Supplementary Figure S4B), and worse performance at both the beginning and the end of the rotarod assay (Supplementary Figure S4C,D).

### NFL Levels Are Increased in the Cerebrospinal Fluid of SCA3 Mice

The degeneration of neurons is believed to lead to an increase in the levels of NFL in patient biofluids, making it a useful biomarker for neurodegenerative diseases (Gaiottino et al., 2013; Zeitlberger et al., 2018; Siller et al., 2019; Weishaupl et al., 2019). In SCA3, we and others have observed that NFL levels in the plasma and CSF can distinguish patients from controls (Li et al., 2019; Peng et al., 2020; Prudencio et al., 2020; Wilke et al., 2020; Peng et al., 2021), and some studies have detected a significant correlation between serum NFL levels and disease severity (Li et al., 2019; Peng et al., 2020; Wilke et al., 2020). Levels are also increased in asymptomatic expanded *ATXN3* carriers compared to healthy controls and may further increase as patients near their anticipated age of onset (Peng et al., 2020; Prudencio et al., 2020; Wilke et al., 2020; Peng et al., 2021). In our mice, we found that NFL was increased in the CSF of Q84 mice compared to Q28 controls (Figure 2H).

### PolyQ-Expanded ATXN3 Accumulates in the Brains of SCA3 Mice

**FIGURE 3:**
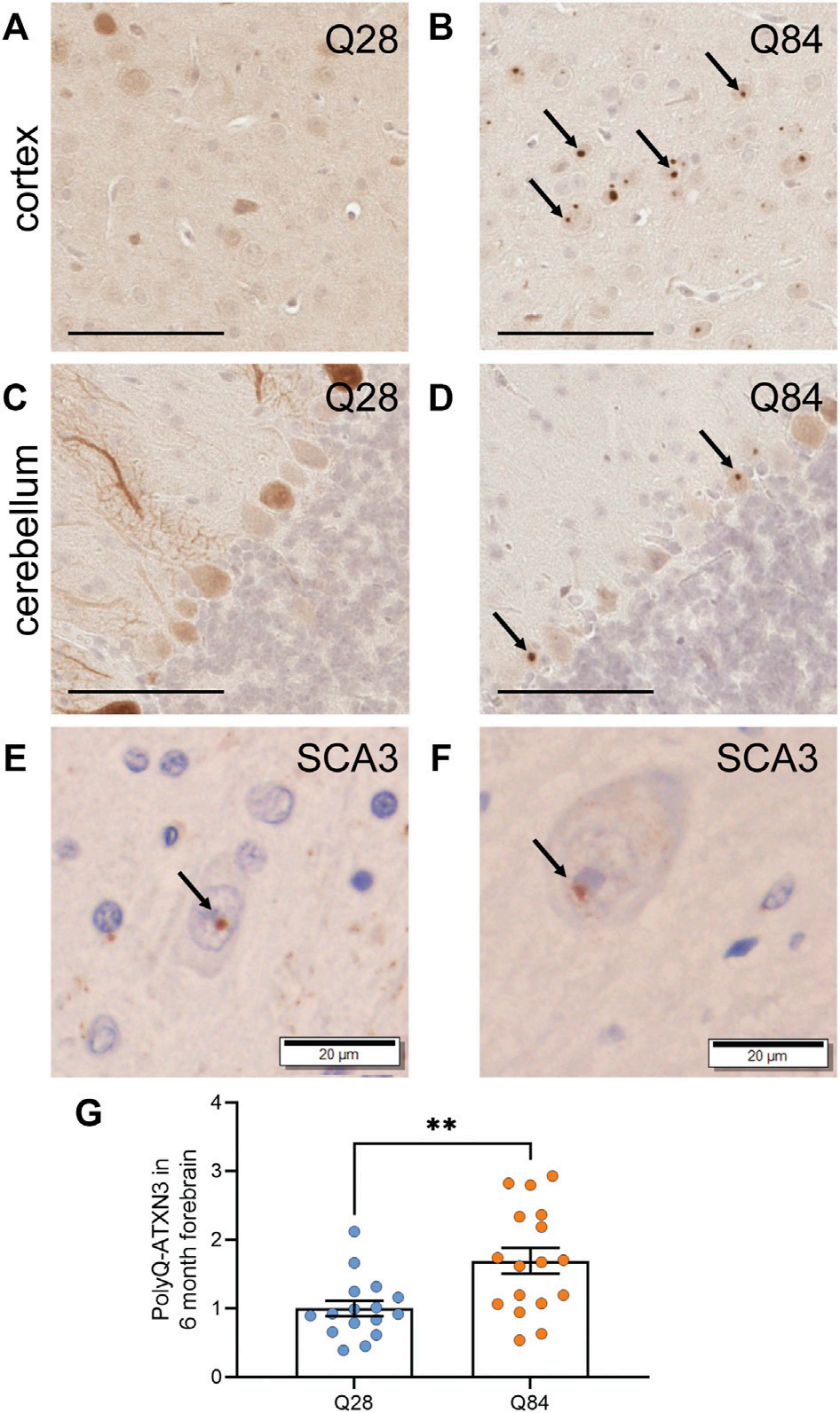
PolyQ-expanded ATXN3 forms inclusions in SCA3 mice **(A**–**D)** Representative images of IHC staining for ATXN3 in the cortex **(A**–**B)** and cerebellum **(C**–**D)** of Q28 **(A**,**C)** and Q84 **(B**,**D)** mice at 6 months of age. Arrows mark inclusions. Scale bars are 100 µm **(E)** IHC staining for ubiquitin in the brain of a SCA3 patient. Arrow marks an inclusion. Scale bar is 20 µm. **(F)** IHC staining for the polyQ antibody clone 1C2 in the brain of a SCA3 patient. Arrow marks an inclusion. Scale bar is 20 µm. **(G)** PolyQ-ATXN3 levels as measured by immunoassay in the forebrain of Q28 and Q84 mice at 6 months of age. Similar results were seen at 3 months; see Supplementary Figure S5. Error bars are the SEM. **p ≤ 0.01 (unpaired *t* test).

A hallmark of polyQ diseases is the accumulation of the expanded protein into proteinaceous inclusions in neurons. For SCA3, polyQ-ATXN3 inclusions are primarily nuclear and positive for ubiquitin. We used immunohistochemistry (IHC) to detect human ATXN3 in the brains of the Q84 and Q28 mice and detected inclusions specifically in the neurons of the Q84 mice at 3 (Supplementary Figure S5A-D) and 6 months of age (Figures 3A–D). Inclusions were observed in both the cortex (Figure 3B and Supplementary Figure S5B) and the cerebellar Purkinje cells (Figure 3D and Supplementary Figure S5D). These inclusions resemble those seen in SCA3 patients (Figures 3E,F), in that they were positive for ubiquitin (Supplementary Figures S6A,B, E,F) and could be detected using the common polyQ antibody clone 1C2 (Supplementary Figures S6C,D, G,H). This result is also consistent with phenotypes seen in other SCA3 mouse models (Ramani et al., 2015; Switonski et al., 2015; Haas et al., 2021), and with rare reports of inclusions in the cortex and Purkinje cells of SCA3 patients (Wilke et al., 2020). We also used our previously published immunoassay to measure the levels of polyQ-ATXN3 in the forebrain (Prudencio et al., 2020; Koike et al., 2021); this assay uses a capture antibody that is believed to target the pathogenic polyQ expansion (Miller et al., 2011). Expression of polyQ-ATXN3 was higher in the forebrain of the Q84 animals compared to controls (Figure 3G and Supplementary Figure S5E). Similar results were seen by Western blot: the ATXN3 protein was detected as high molecular weight species when we analyzed forebrain lysates from Q84 animals, but not when we used lysates from Q28 controls (Supplementary Figures S7A-C). The relative expression of the ATXN3 monomer in each mouse model reflected relative differences in transgene levels as measured by qPCR (Supplementary Figures S7B,E and Supplementary Figure S1A). Unfortunately, polyQ-ATXN3 levels in the cerebellum were too low to be detected above background using our immunoassay and there was little high molecular weight species observed on Western blots from this brain region (Supplementary Figure S7D), mirroring our observation that inclusions were limited to the Purkinje cells in the cerebellum and comparatively less abundant than in the cortex. Human *ATXN3* transgene levels were also lower in the cerebellum than in the forebrain, although still detectable by qPCR (Supplementary Figure S1A); similarly, endogenous *Atxn3* RNA levels are slightly lower in the cerebellum than in the forebrain in both Q28 and Q84 animals (Supplementary Figure S1B). The decreased cerebellar polyQ-ATXN3 protein abundance we observed could also be influenced by neuronal loss in this region, as the expanded protein appears to be particularly toxic to Purkinje cells.

### SCA3 Mice Develop pTDP-43 Inclusions

**FIGURE 4:**
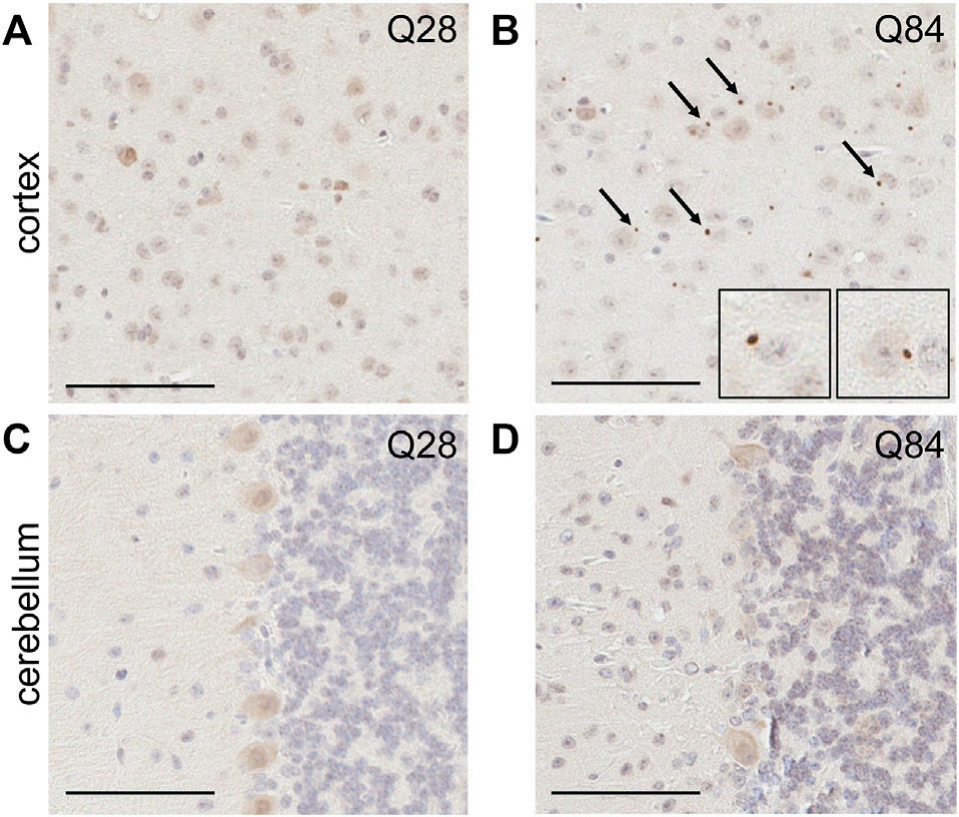
SCA3 mice develop pTDP-43 inclusions in the cortex. Representative images of IHC staining for pTDP-43 in the cortex **(A**–**B)** and cerebellum **(C**–**D)** of Q28 **(A**,**C)** and Q84 **(B**,**D)** mice at 6 months of age. Similar results seen at 3 months; see Supplementary Figure S8. Arrows mark inclusions. Insets in **(B)** are higher magnification images of inclusions. Scale bars are 100 µm.

The presence of inclusions containing phosphorylated TAR DNA-binding protein 43 (pTDP-43) is a hallmark of several neurodegenerative diseases. Such TDP-43 proteinopathy has not traditionally been associated with polyQ diseases, but inclusions have in fact been reported in SCA3 (Tan et al., 2009; Seidel et al., 2010), SCA2 (Toyoshima et al., 2011; Mori et al., 2014) and Huntington’s disease patients (Schwab et al., 2008; St-Amour et al., 2018; Sanchez et al., 2021). To determine whether our model could recapitulate this observation, we used IHC to evaluate the burden of pTDP-43 inclusions in the brains of our Q84 and Q28 mice. Interestingly, pTDP-43 inclusions accumulated in the cortex of the Q84 animals (Figure 4B and Supplementary Figure S8B), but not in the Q28 animals (Figure 4A and Supplementary Figure S8A). To our knowledge, this is the first report of pTDP-43 proteinopathy in a SCA3 mouse model. Of note, pTDP-43 inclusions were not observed in the cerebellar Purkinje cells (Figure 4D and Supplementary Figure S8D), but this result is expected as pTDP-43 proteinopathy is not observed in this region in patients (Tan et al., 2009; Seidel et al., 2010).

### PolyQ-Expanded ATXN3 can Be Detected in the Plasma of SCA3 Mice and Levels Correlate With Disease Features

**FIGURE 5:**
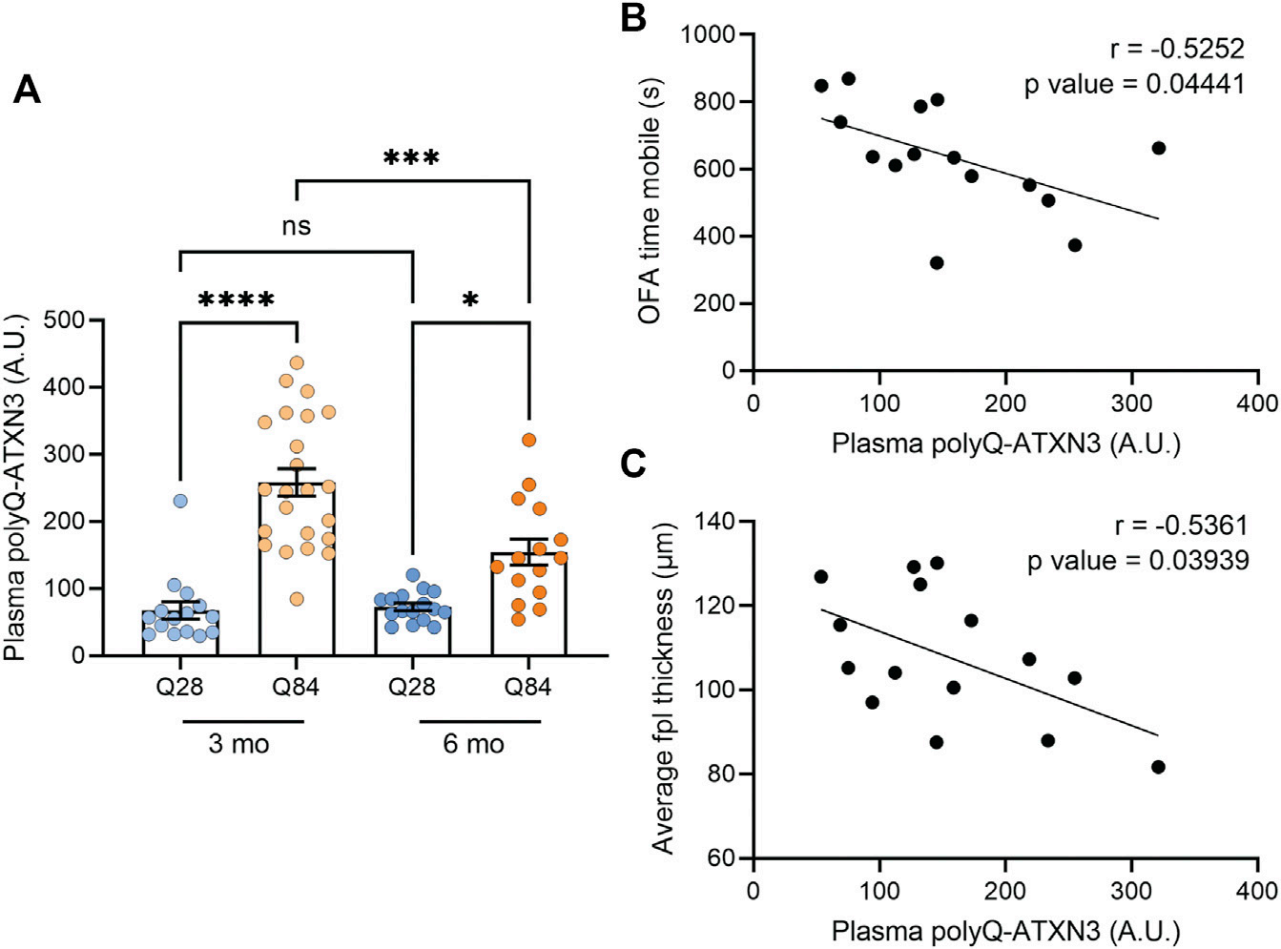
Higher polyQ-ATXN3 levels correlate with decreased mobility and a thinner molecular layer in SCA3 mice. **(A)** PolyQ-ATXN3 levels as measured by immunoassay in the plasma of Q28 and Q84 mice at 3 and 6 months of age. Error bars are the SEM. *p ≤ 0.05, **p ≤ 0.01, ***p ≤ 0.001, ****p ≤ 0.0001, ns = non-significant (1-way ANOVA with Tukey’s multiple comparisons test). **(B)** Correlation analysis between plasma polyQ-ATXN3 levels and the time spent mobile in the open field assay for 6-month-old Q84 mice. Pearson’s correlation coefficient (r) and *p* values are indicated. The line represents an estimated simple linear regression. **(C)** Correlation analysis between plasma polyQ-ATXN3 levels and the cerebellar molecular layer thickness at the posterolateral fissure (fpl) for 6-month-old Q84 mice. Pearson’s correlation coefficient (r) and *p* values are indicated. The line represents an estimated simple linear regression.

We recently demonstrated that our polyQ-ATXN3 immunoassay can detect the expanded protein in the plasma, CSF (Prudencio et al., 2020) and urine (Koike et al., 2021) of SCA3 patients, and we found that the level of the mutant protein in these biofluids accurately distinguishes SCA3 patients from healthy and disease controls (Prudencio et al., 2020; Koike et al., 2021). Elevated polyQ-ATXN3 levels in the urine also associated with an earlier age of onset (Koike et al., 2021). We were unable to obtain enough CSF from each mouse to measure both NFL and polyQ-ATXN3 in the same cohort. Nevertheless, we used our immunoassay to measure polyQ-ATXN3 levels in the plasma of the mice and detected an increase in polyQ-ATXN3 specifically in the Q84 animals compared to controls (Figure 5A). We then performed correlation analyses to determine whether polyQ-ATXN3 levels in the plasma were associated with any disease-related phenotypes in 6-month-old mice. We detected a slight but significant inverse correlation between the plasma polyQ-ATXN3 levels in Q84 mice and the time spent mobile in the open field assay: animals with higher plasma polyQ-ATXN3 levels were less mobile (Figure 5B). There was also a correlation between polyQ-ATXN3 levels and the thickness of the cerebellar posterolateral fissure at this age (Figure 5C).

## Discussion

In the present study we show that the AAV-mediated expression of an expanded 84-repeat ATXN3 construct results in behavioral deficits in mice at both 3 and 6 months of age. The mice showed impaired performance in a range of behavioral tests used to assess ataxia in rodents, suggesting they recapitulate the locomotor, coordination and balance defects associated with human spinocerebellar ataxias like SCA3. These phenotypes were observed in both male and female mice, without any apparent sex-based effects. Interestingly, our mice also showed selective neuronal vulnerability that is reminiscent of human ataxia: we observed a mild yet significant decrease in cerebellar Purkinje cells in the Q84 animals but did not observe any differences in cortical neuronal number, even when we focused on the motor cortex alone. While it is tempting to suspect that these differences in neuronal loss are due to regional differences in mutant *ATXN3* expression, the RNA levels, protein inclusions, and polyQ-ATXN3 protein levels were all comparatively higher in the forebrain of our mice than in the cerebellum. We therefore conclude that the cerebellar neurons were simply more susceptible to polyQ-ATXN3-induced toxicity than other neuronal subtypes. The relatively lower levels of polyQ-ATXN3 observed in the cerebellum could also be a reflection of this cerebellar-specific polyQ-ATXN3 toxicity: we saw a similar effect in a separate mouse model where the expression of a highly toxic protein appeared to taper off over time as a result of acute neuronal loss (Zhang et al., 2019). Importantly, as is consistent with our model, cortical neurons are also generally spared in human SCA3, with only mild changes in cortical atrophy and cognitive dysfunction noted in some patients (Yamada et al., 2001; Lopes et al., 2013). It is worth noting, however, that Purkinje cell loss, while occasionally observed (Scherzed et al., 2012), is not as prominent in SCA3 as it is in other spinocerebellar ataxias (Sachdev et al., 1982; Yuasa et al., 1986; Takiyama et al., 1994; Durr et al., 1996). It is unclear why this is the case, or why our mouse model, like other SCA3 mouse models (Cemal et al., 2002; Bichelmeier et al., 2007; Boy et al., 2010; Switonski et al., 2015; Haas et al., 2021), still shows some vulnerability in these cells. Indeed, the reasons underlying patterns of selective vulnerability in most neurodegenerative diseases are not understood, but an enticing hypothesis in the field of polyQ disease research is that the endogenous functions of the host protein influence its effects on different cell types. While the polyQ expansion increases the aggregation propensity of the mutant protein, it is also believed to alter its native activity and protein-protein interactions (Orr, 2012; McLoughlin et al., 2020). ATXN3 is a deubiquitinating enzyme (Burnett et al., 2003; McLoughlin et al., 2020), and a general increase in ubiquitinated proteins is observed in both *Atxn3* knockout mice (Schmitt et al., 2007) and SCA3 patients (Sittler et al., 2018). The protein is also believed to play a role in transcription (Li et al., 2002), and changes in the transcriptome are seen in SCA3 mice (Chou et al., 2008) and patient samples (Evert et al., 2003). These transcriptomic changes occur at late stages in mice, but even presymptomatic mice show changes in total and phosphorylated protein levels (Wiatr et al., 2019). Together, these effects on gene and protein expression could impact the function of individual neuronal subtypes in unique ways. For example, mouse studies suggest that the expression of the polyQ-expanded ATXN3 protein increases the intrinsic excitability of Purkinje cells (Shakkottai et al., 2011), rendering them more vulnerable to excitotoxic stress. This change in excitability was attributed to changes in the expression of voltage-gated potassium channels in these cells (Bushart et al., 2021). We observed similar changes in our mice: the RNA expression of the voltage-gated potassium channel *Kcnc3* was decreased in the cerebellum of Q84 mice (Supplementary Figure S9A), but not in the forebrain (Supplementary Figure S9B).

While research mostly focuses on polyQ-ATXN3 protein inclusions in SCA3, we also observed pTDP-43 inclusions in the cortex of our Q84 mice. TDP-43 pathology is generally associated with ALS, frontotemporal dementia (FTD), and other TDP-43 proteinopathies, and yet, there is evidence that TDP-43 may also be mislocalized and aggregated in polyQ disorders. Rare TDP-43 inclusions have been observed in the lower motor neurons and axonal tracts of SCA3 patients but were not detected in upper motor neurons (Tan et al., 2009; Seidel et al., 2010). TDP-43 inclusions have also been observed in SCA2, although in this case, inclusions were detected in several regions of the central nervous system except for the lower motor neurons (Toyoshima et al., 2011; Mori et al., 2014). Nevertheless, although the regional distribution of inclusions is different in these diseases, both show TDP-43 inclusions that morphologically resemble the skein-like inclusions observed in ALS. The inclusions we see in our Q84 mice are round and perinuclear, and in this way they resemble the pTDP-43 inclusions seen in our ALS mouse models (Chew et al., 2015; Chew et al., 2019). Whether these round inclusions also contribute to SCA3 pathogenesis remains to be determined, but it is reasonable to predict that both these inclusions and the skein-like inclusions seen in patients negatively impact TDP-43 activity, and this could be detrimental to neurons. TDP-43 pathology has also been linked to Huntington’s disease, although in this disease TDP-43 and the polyQ-expanded protein sometimes co-localize (Schwab et al., 2008; St-Amour et al., 2018)—a feature not seen in SCA2 (Toyoshima et al., 2011; Mori et al., 2014) or SCA3 (Tan et al., 2009; Seidel et al., 2010). Work in cell culture suggests that TDP-43 is sequestered into both pure polyQ aggregates and polyQ-expanded huntingtin inclusions, indicating a potential interaction between these two aggregation-prone proteins (Fuentealba et al., 2010). Co-localization was not observed in a more recent report on Huntington’s disease, however, arguing for further future analysis of this phenotype (Sanchez et al., 2021). Overall, these findings are intriguing, but the differences between the presentation of TDP-43 inclusions in these different polyQ disorders may indicate that TDP-43 pathology is induced by separate pathways in each case. Our study is the first report of pTDP-43 proteinopathy in a SCA3 mouse model and our Q84 mice could be used in future studies aimed at understanding the mechanisms underlying pTDP-43 inclusion formation in SCA3 and its effects (if any) on disease features. It will also be interesting to see if this proteinopathy is observed in other SCA3 animal models, as, to our knowledge, this has yet to be tested.

The fact that our AAV-mediated SCA3 mouse model mimics various disease features is promising, but it is by no means the first or only mouse model to recapitulate SCA3. Several other SCA3 mouse models have been developed over the years, using transgenic overexpression (Ikeda et al., 1996; Cemal et al., 2002; Bichelmeier et al., 2007; Chou et al., 2008; Boy et al., 2010; Silva-Fernandes et al., 2010; Silva-Fernandes et al., 2014), knock-in (Ramani et al., 2015; Switonski et al., 2015; Haas et al., 2021), and conditional expression schemes (Boy et al., 2009). Some studies have also used lentivirus to express the expanded ATXN3 protein in specific regions of the adult rodent brain, such as the cerebellum (Nobrega et al., 2013a) or the striatum (Alves et al., 2008; Simoes et al., 2012). Like our model, these SCA3 mouse models also show behavioral deficits, neuronal inclusions, and neuronal dysfunction that sometimes leads to cerebellar cell loss. The advantage of our model therefore stems from its utility. The use of AAV technology in mouse modelling allows for the generation of disease-relevant animal models without the necessity to maintain specific mouse lines, and the injection protocol we use allows for the expression of the gene of interest throughout the brain, resulting in phenotypes that resemble those seen in more traditional knock-in or transgenic models. AAV technology also facilities studies aimed at exploring potential genetic modifiers or testing mechanistic hypotheses: our *ATXN3* AAV constructs can be directly injected into pre-existing mutant or knockout mouse lines or combined with CRISPRi technology to easily and efficiently determine whether altering the expression of a given gene of interest could influence disease outcomes. Indeed, some studies have already used a lentiviral model of SCA3 to assess whether lowering endogenous *Atxn3* levels or altering other factors can influence the effects of mutant ATXN3 on the striatum (Simoes et al., 2012; Goncalves et al., 2013; Cunha-Santos et al., 2016; Nobre et al., 2021); we believe future studies using our model will build upon this work. Furthermore, our observation that the potential biomarkers NFL and polyQ-ATXN3 are elevated in the biofluids of our mice also supports the use of our model in future preclinical studies. We believe this new mouse model will prove to be a great asset to the field of SCA3 research.

The observation that CSF NFL and plasma polyQ-ATXN3 levels are elevated in our Q84 mouse model compared to the Q28 controls is in accordance with what we and others have observed in SCA3 patient samples, and with a previous study that looked at NFL levels in cerebellar extracts from SCA3 mice (Costa et al., 2020). NFL is believed to be released from neurons as they degenerate, resulting in its accumulation in biofluids. Increases in NFL are therefore considered a marker of neuronal injury and are predicted to be elevated in neurodegenerative diseases. NFL levels are elevated in both the blood and CSF of SCA3 patients and can accurately distinguish patients from controls (Li et al., 2019; Peng et al., 2020; Prudencio et al., 2020; Wilke et al., 2020; Peng et al., 2021). Blood NFL levels have also been found to associate with disease severity (Li et al., 2019; Peng et al., 2020; Wilke et al., 2020) and some studies suggest it could potentially be used as an indicator of disease progression and potential onset in asymptomatic expanded *ATXN3* carriers (Wilke et al., 2020; Peng et al., 2021). PolyQ-ATXN3 can also be detected in the blood and CSF of SCA3 patients using immunoassays, and it is elevated in patients compared to controls (Prudencio et al., 2020; Hubener-Schmid et al., 2021). Our immunoassay to detect polyQ-ATXN3 makes use of previously described polyQ antibody (clone 3B5H10) that is predicted to bind a lower molecular weight, toxic oligomeric form of the protein sequence (Miller et al., 2011). The expression of this protein conformer predicted cell death in a polyQ cell model (Miller et al., 2011). Therefore, the accumulation of this conformer in patient biofluids could also potentially be a marker of polyQ-induced neuronal degeneration. While we did not detect any correlations between CSF or plasma polyQ-ATXN3 levels and disease features in patients in our initial study (Prudencio et al., 2020), we did detect an association between urine polyQ-ATXN3 levels and an earlier age of onset in a follow-up report (Koike et al., 2021). A separate study using a slightly different immunoassay noted a correlation between plasma polyQ-ATXN3 levels and disease severity, but also failed to see a correlation between CSF polyQ-ATXN3 levels and disease features (Hubener-Schmid et al., 2021). In our 6-month-old Q84 animals, we found that higher plasma polyQ-ATXN3 levels correlated with a thinner molecular layer thickness at the cerebellar posterolateral fissure and a decrease in the overall time the mice spent mobile in the open field assay. Plasma polyQ-ATXN3 levels did not correlate with polyQ-ATXN3 levels in the forebrain in the Q84 mice (Supplementary Figure S10), and this is perhaps consistent with human studies showing that plasma polyQ-ATXN3 levels do not associate with levels in the CSF (Prudencio et al., 2020; Hubener-Schmid et al., 2021). Overall, while preliminary, our findings suggest that plasma polyQ-ATXN3 levels can correlate with disease features in mice and warrant its further investigation as a biomarker in human SCA3.

In conclusion, we have developed and characterized an AAV-based mouse model of SCA3 that exhibits ataxia-like defects in locomotor behavior, mutant ATXN3 and pTDP-43 inclusion pathology, cerebellar-specific neuronal degeneration, elevated CSF NFL levels, and increased plasma polyQ-ATXN3 levels. The use of AAV technology in this model will facilitate future mechanistic studies, while the observation that the mice mimic expected changes in potential biomarkers will bolster future preclinical trials. Finally, our observation that elevated plasma polyQ-ATXN3 levels correlated with measures of cerebellar degeneration and locomotion in 6-month-old mice supports the use of our polyQ-ATXN3 immunoassay as a biomarker in SCA3.

## Data Availability

The original contributions presented in the study are included in the article/Supplementary Material, further inquiries can be directed to the corresponding authors.

## Abbreviations

SCA3, spinocerebellar ataxia type 3; ATXN3, ataxin-3; polyQ, polyglutamine; NFL, neurofilament light; CSF, cerebrospinal fluid; AAV, adeno-associated virus; IHC, immunohistochemistry; TDP-43, TAR DNA-binding protein 43.
