# Supplementary material for: Plasma PolyQ-ATXN3 Levels Associate With Cerebellar Degeneration and Behavioral Abnormalities in a New AAV-Based SCA3 Mouse Model
Source: Front Cell Dev Biol. 2022 Mar 21;10:863089. doi: 10.3389/fcell.2022.863089 (PMC8977414; doi:10.3389/fcell.2022.863089)
Supplement: Supplementary file 1 [file DataSheet1.pdf]

Supplementary Material

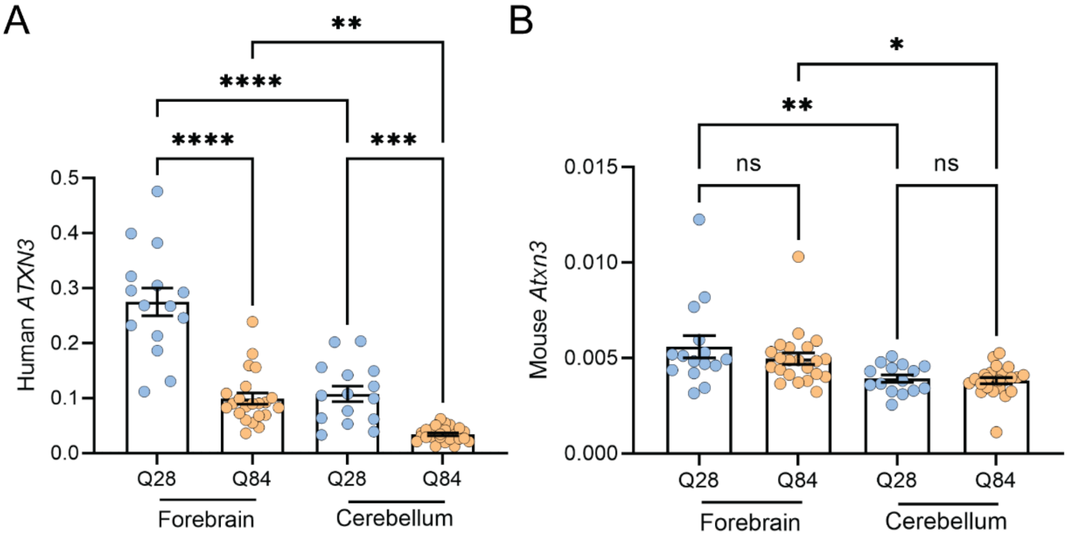

Supplementary Figure S1: Human *ATXN3* is expressed in the mice and endogenous mouse *Atxn3* levels did not change. (**A**) qPCR analysis of human *ATXN3* RNA levels in the forebrain and cerebellum of 3-month-old Q28 and Q84 animals. (**B**) qPCR analysis of mouse *Atxn3* RNA levels in the forebrain and cerebellum of 3-month-old Q28 and Q84 animals. For both panels, forebrain and cerebellar samples were run on the same plate for comparison of regional levels and all values were corrected for background signal. Mouse *Gapdh* was used as an endogenous control. Error bars are the standard error of the mean (SEM). *$p \leq 0.05$, ** $p \leq 0.01$, ***$p \leq 0.001$, ****$p \leq 0.0001$, ns = non-significant (1-way ANOVA with Tukey's multiple comparisons test).

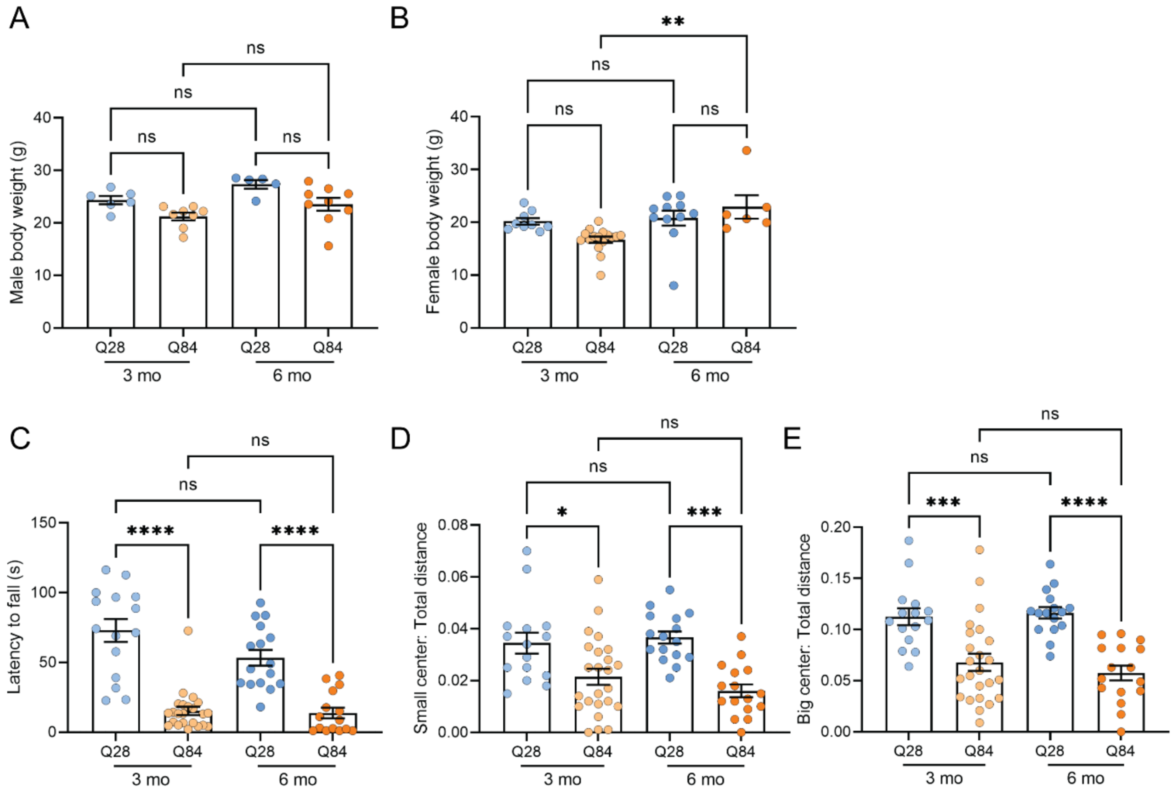

Supplementary Figure S2: SCA3 mice showed normal body weights and ataxia-like behavioral defects. (**A-B**) Average body weight (grams) of male (**A**) and female (**B**) Q28 and Q84 animals at 3 and 6 months of age. (**C**) Latency to fall (seconds) on the wire hang assay for Q28 and Q84 animals at 3 and 6 months of age. (**D**) Ratio of distance traveled in the small "center zone" versus total distance traveled during the open field assay for Q28 and Q84 animals at 3 and 6 months of age. (**E**) Ratio of distance traveled in the big "center zone" versus total distance traveled during the open field assay for Q28 and Q84 animals at 3 and 6 months of age. For all panels, error bars are the SEM. *$p \leq 0.05$, **$p \leq 0.01$, ***$p \leq 0.001$, ****$p \leq 0.0001$, ns = non-significant (1-way ANOVA with Tukey's multiple comparisons test).

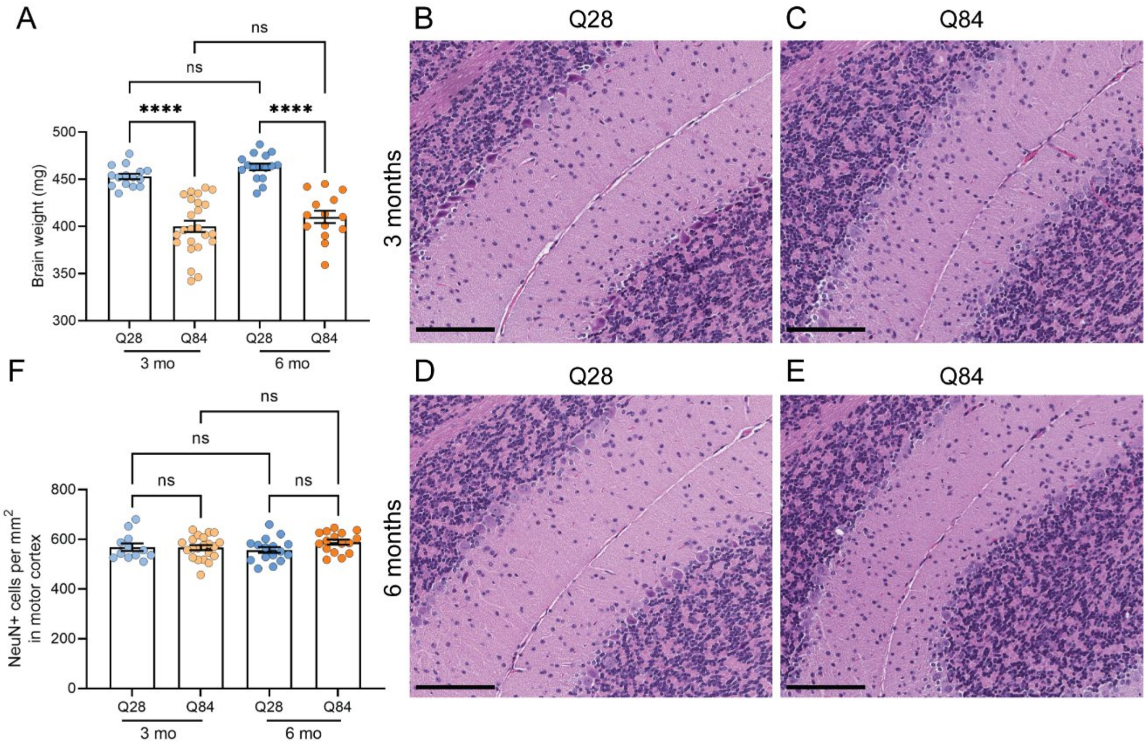

Supplementary Figure S3: SCA3 mice show decreased brain weight and a thinner molecular layer thickness, but no neuronal loss in the motor cortex. (**A**) Average brain weight (micrograms) at harvest in Q28 and Q84 animals at 3 and 6 months of age. (**B**-**E**) Representative images of the posterolateral fissure in the cerebellum of Q28 (**B**, **D**) and Q84 (**C**, **E**) mice at 3 (**B**-**C**) and 6 months (**D**-**E**) of age. The molecular layer is thinner in the Q84 animals. Slides are stained with H&E. Scale bars are 100 µm. (**F**) Number of NeuN-positive cells per mm^2 in the motor cortex of Q28 and Q84 animals at 3 and 6 months of age. For all panels, error bars are the SEM. ****$p \leq 0.0001$, ns = non-significant (1-way ANOVA with Tukey's multiple comparisons test).

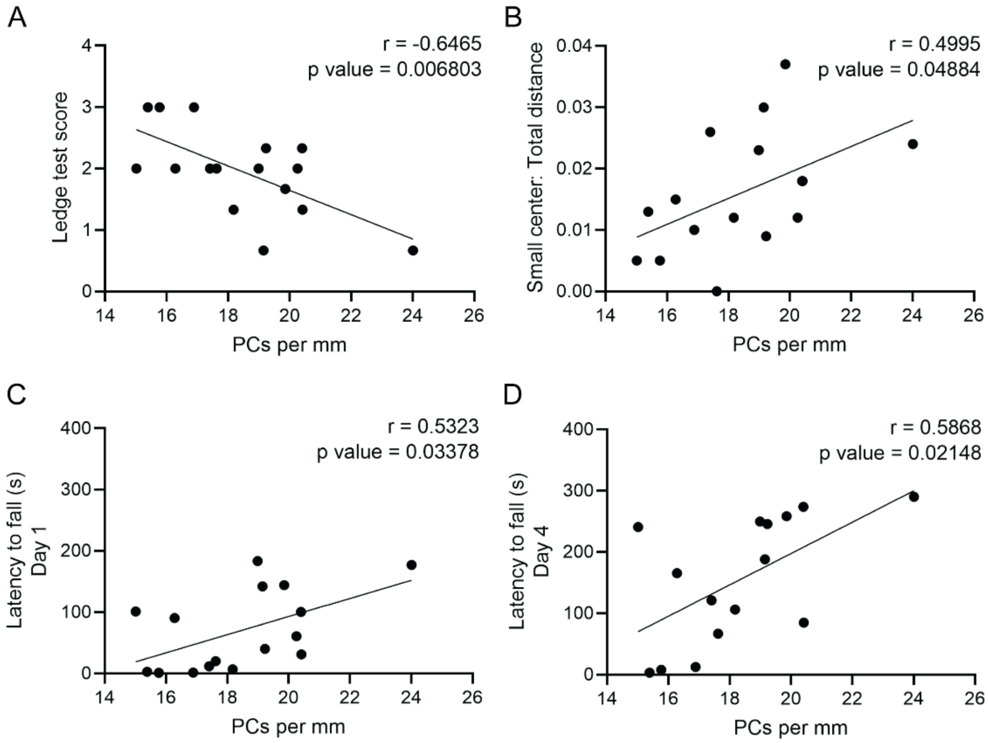

Supplementary Figure S4: The number of Purkinje cells in Q84 mice correlate with behavioral phenotypes. (**A-D**) Correlation analyses between the number of cerebellar Purkinje cells (PCs) per millimeter in Q84 mice and their score in the ledge test assay (**A**), small center to total distance ratio in the OFA assay (**B**), or latency to fall (seconds) in the rotarod assay at either Day 1 (**C**) or Day 4 (**D**). For all panels, Pearson's correlation coefficient (r) and p values are indicated. The lines represent an estimated simple linear regression.

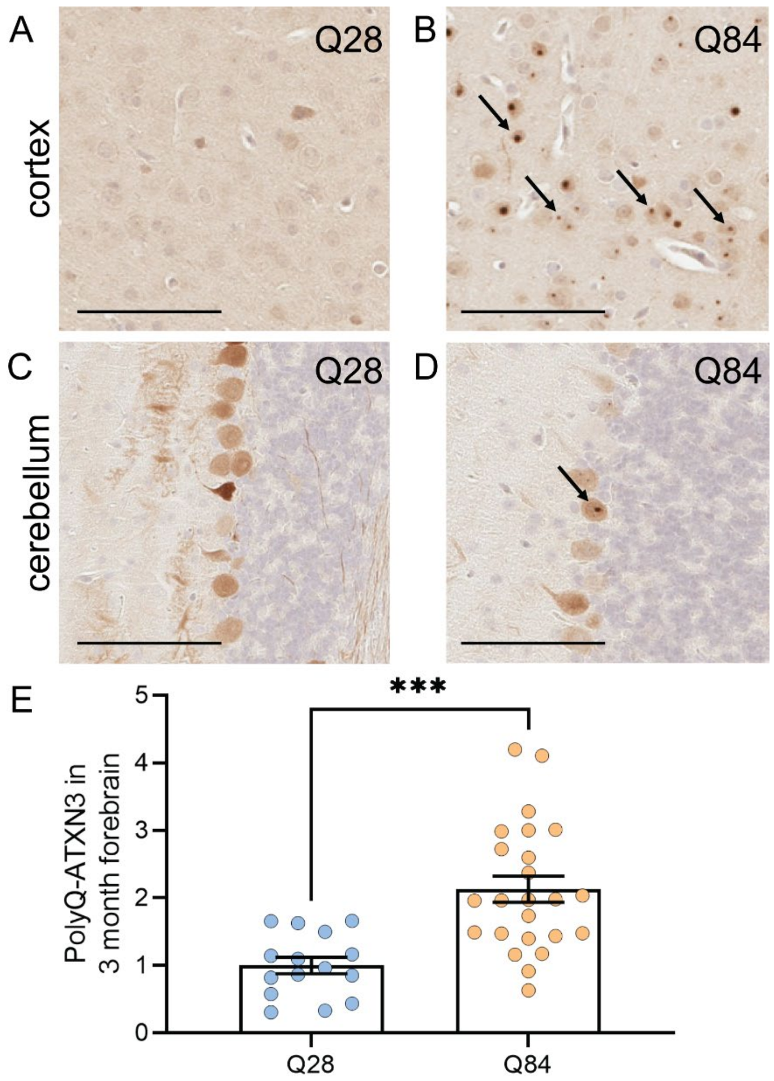

Supplementary Figure S5: PolyQ-expanded ATXN3 forms inclusions in SCA3 mice at 3 months of age. (**A-D**) Representative images of IHC staining for ATXN3 in the cortex (**A**-**B**) and cerebellum (**C**-**D**) of Q28 (**A**, **C**) and Q84 (**B**, **D**) mice at 3 months of age. Arrows mark inclusions. Scale bars are 100 µm. (**E**) PolyQ-ATXN3 levels as measured by immunoassay in the forebrain of Q28 and Q84 mice at 3 months of age. Error bars are the SEM. ***$p \leq 0.001$ (unpaired *t* test).

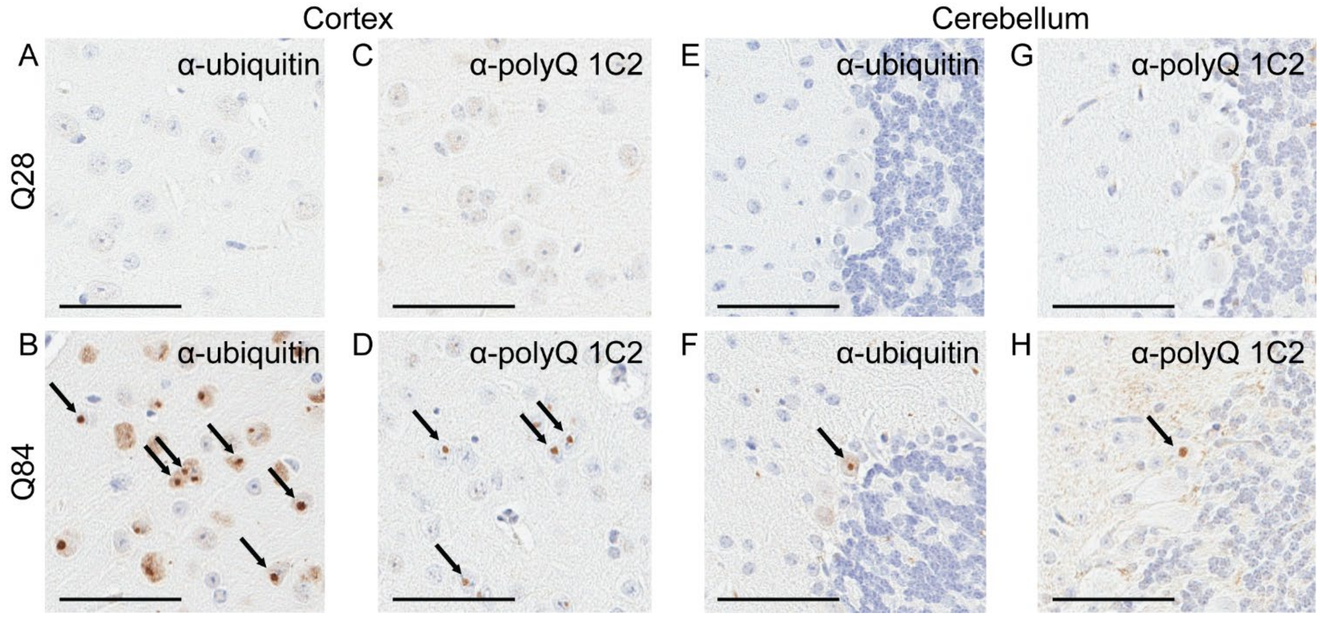

Supplementary Figure S6: PolyQ-expanded ATXN3 inclusions in SCA3 mice are also positive for ubiquitin and the 1C2 polyQ epitope. Representative images of IHC staining for ubiquitin (**A**-**B**, **E**-**F**) and the polyQ antibody clone 1C2 (**B**-**D**, **G**-**H**) in the cortex (**A**-**D**) and cerebellum (**E**-**H**) of Q28 (**A**, **C**, **E**, **G**) and Q84 (**B**, **D**, **F**, **H**) mice. Images are from 3-month-old animals. Arrows mark inclusions. Scale bars are 100 µm.

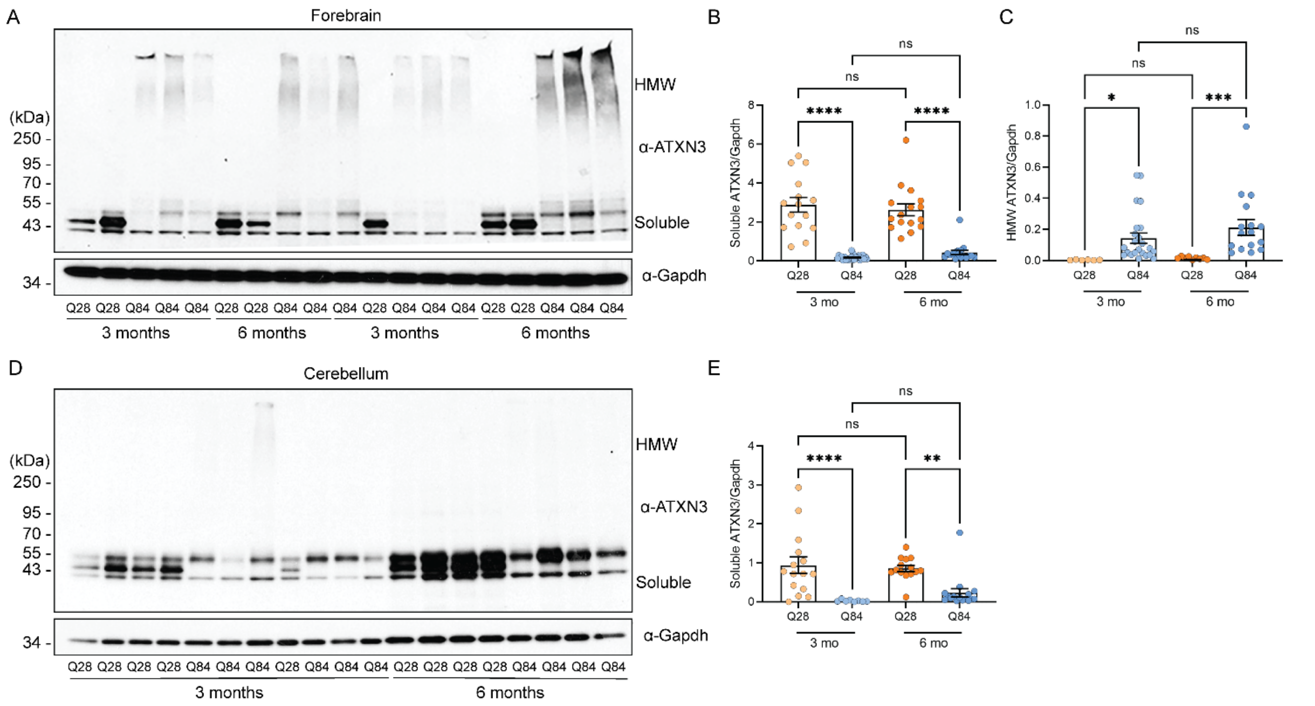

Supplementary Figure S7: Q84 mice show increased high molecular weight ATXN3 upon Western blot analyses. Representative immunoblots of forebrain (**A**) and cerebellar (**D**) lysates from the indicated Q28 and Q84 mice using an anti-ATXN3 antibody. Gapdh was used as a loading control. (**B**) Densitometric analysis of the ATXN3 monomer compared to Gapdh in the forebrain. (**C**) Densitometric analysis of high molecular weight (HMW) ATXN3 compared to Gapdh in the forebrain. (**E**) Densitometric analysis of the ATXN3 monomer compared to Gapdh in the cerebellum. For all panels, error bars are the SEM. *$p \leq 0.05$, **$p \leq 0.01$, ***$p \leq 0.001$, ****$p \leq 0.0001$, ns = non-significant (1-way ANOVA with Tukey's multiple comparisons test).

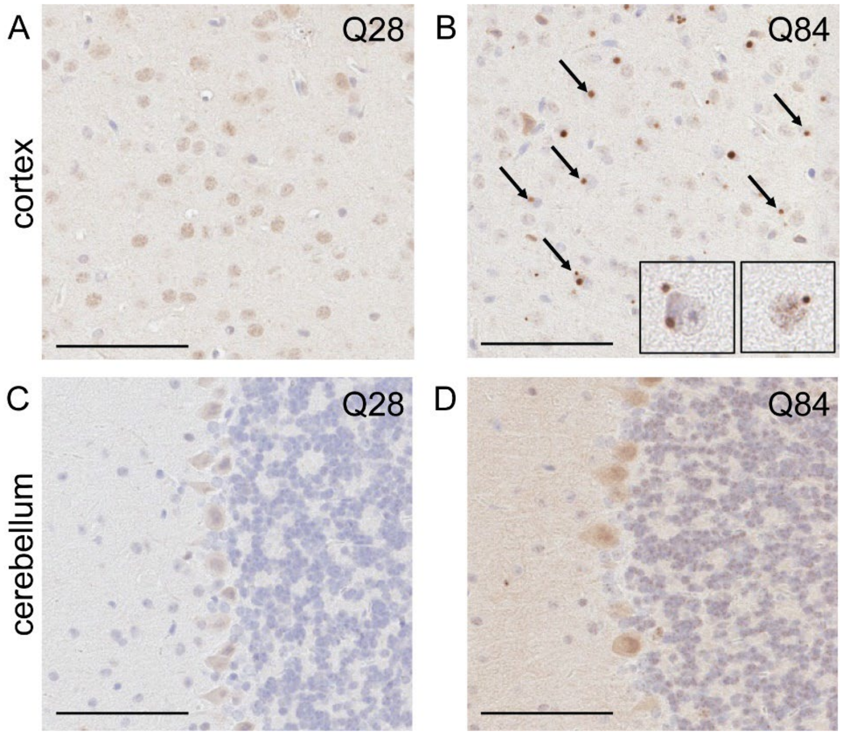

Supplementary Figure S8: pTDP-43 inclusions are observed in the cortex of SCA3 mice at 3 months of age. Representative images of IHC staining for pTDP-43 in the cortex (**A**-**B**) and cerebellum (**C**-**D**) of Q28 (**A**, **C**) and Q84 (**B**, **D**) mice at 3 months of age. Arrows mark inclusions. Insets in (**B**) are higher magnification images of inclusions. Scale bars are 100 µm.

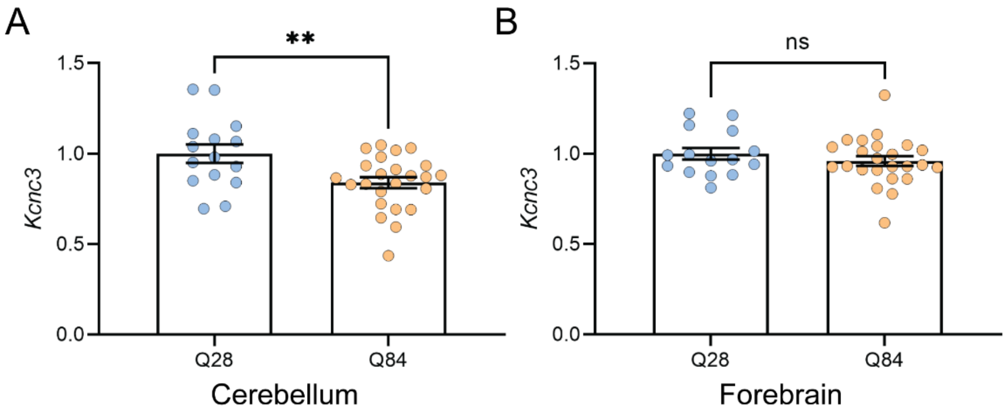

Supplementary Figure S9: *Kcnc3* expression is decreased in the cerebellum of SCA3 mice but not in the forebrain. (**A**-**B**) qPCR analysis of mouse *Kcnc3* RNA levels in the cerebellum (**A**) and forebrain (**B**) of 3-month-old Q28 and Q84 animals. Mouse *Gapdh* was used as an endogenous control and values were normalized against the average levels in Q28 animals. Error bars are the SEM. $^{**}p \leq 0.01$, ns = non-significant (unpaired *t* test).

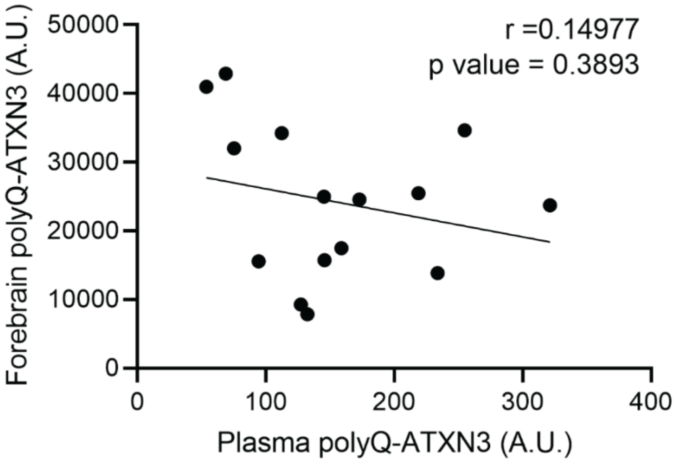

Supplementary Figure S10: polyQ-ATXN3 levels in the plasma do not correlate with levels in the forebrain of Q84 mice. Correlation analysis between plasma polyQ-ATXN3 levels and forebrain polyQ-ATXN3 levels in 6-month-old Q84 mice. Pearson's correlation coefficient (r) and p values are indicated. The line represents an estimated simple linear regression. No correlation was observed between these two factors.
